# A Public Data Set With Ground Reaction Forces of Human Balance in Individuals With Parkinson's Disease

**DOI:** 10.3389/fnins.2022.865882

**Published:** 2022-04-19

**Authors:** Claudia Eunice Neves de Oliveira, Caroline Ribeiro de Souza, Renata de Castro Treza, Sandy Mikie Hondo, Emanuele Los Angeles, Claudionor Bernardo, Thiago Kenzo Fujioka Shida, Luana dos Santos de Oliveira, Thayna Magalhães Novaes, Débora da Silva Fragoso de Campos, Emerson Gisoldi, Margarete de Jesus Carvalho, Daniel Boari Coelho

**Affiliations:** ^1^Department of Biomedical Engineering, Federal University of ABC, São Bernardo do Campo, Brazil; ^2^Center for Mathematics, Computation and Cognition, Federal University of ABC, São Bernardo do Campo, Brazil; ^3^School of Physical Education and Sport, University of São Paulo, São Paulo, Brazil; ^4^Ambulatório de Distúrbios de Movimento, Faculdade de Medicina do ABC, Santo André, Brazil

**Keywords:** posture, posturography, biomechanics, motor control, levodopa (L-Dopa)

## Introduction

Parkinson's disease is mainly characterized by clinical motor manifestations, with postural instability being a predominant symptom that leads to many falls. One way in which postural control is assessed is to measure the characteristics of postural sway during quiet standing. In this test, subjects standstill on a force platform, which records ground forces and reaction moments that can be used to calculate the center of pressure (CoP) (Duarte and Freitas, 2010). The CoP is the most common posturographic measure used in the assessment of postural control, and is considered a neuromuscular response to the body's center of mass displacement.

In PD, postural sway can be abnormal long before it is clinically evident and before starting treatment with levodopa medication (Mancini et al., 2011; Stylianou et al., 2011; Nantel et al., 2012). For example, in the early stages of Parkinson's disease, with the Hoehn and Yahr (H&Y) scales being 1, postural sway in PD is hardly detected only in clinical observation, as it is initially subtle and not always very evident (Mancini et al., 2012). However, when measured using more accurate measuring instruments, such as the force platform, there are postural problems in the early stages of PD, demonstrated by an abnormality of sway in patients with mildly symptomatic PD (Beuter et al., 2008; Chastan et al., 2008). Mancini et al. (2012) observed that postural sway measured by the CoP is a biomarker of PD progression. Therefore, quantitative evaluation measures in the different stages of the disease are necessary.

Drug treatment through the administration of levodopa, although effective for most motor symptoms, is controversial for postural control (Baston et al., 2016; Peterson and Horak, 2016). Despite improving some clinical scales such as the UPDRS, the effect of medication on objective measures of postural control is controversial (Chastan et al., 2008). About quiet standing, there is evidence that levodopa causes more significant postural sway, especially in H&Y stages 3 and 4 (Rocchi et al., 2002; Curtze et al., 2015). One possible explanation is levodopa induction of dyskinesia (Chung et al., 2010), which could explain the increase in falls under medication. Although PD patients may reweigh sensory information in response to changes in external stimuli, there is no evidence that levodopa contributes to improving this sensory reweighing in postural control (Feller et al., 2019).

Other public databases on human balance have already been published (Santos and Duarte, 2016; Dos Santos et al., 2017). However, this study is the first public data set in patients with idiopathic PD. This article aims to describe a public data set with a rich quantitative and qualitative assessment of static balance in patients with idiopathic PD through measurements of ground reaction forces (GRF). In addition, we provide relevant data on the clinical condition of the subjects, both in the ON and OFF state of the medication, through the physical status scales: Unified Parkinson's disease rating scale motor aspects of experiences of daily living (UPDRS-II) and motor score (UPDRS-III), Hoehn & Yahr (H&Y), New Freezing of Gait Questionnaire (NFOG-Q), Montreal Cognitive Assessment (MoCA), Mini-Test of Balance Assessment System scale (Mini-BESTest), and Falls Efficacy Scale International (FES-I).

## Methods

The data collection was performed in the Laboratory of Biomechanics and Motor Control at the Federal University of ABC, Brazil. The local ethics committee approved this study, and all patients signed a consent form before the data collection. The patients were in a stable dose of L-DOPA for at least one month. The PD patients participated in two experimental sessions, one of which was in the ON condition of the medication and the other was in the OFF condition. To be considered ON condition, participants had taken dopaminergic medication one hour before starting the session to ensure dose stabilization. In the OFF condition, the participants were at least 12 h without using any medication for Parkinson's disease. The order of the sessions was randomized among the patients. The start time of the experiment was the same as the experimental sessions.

### Participants

A convenience sample of 32 idiopathic PD patients (eight females and 24 males) was recruited to participate in this study. The patients were recruited from local communities and included local neighborhoods and ambulatory movement disorders. The patients were interviewed to collect information about their demographic characteristics, socio-cultural characteristics, and overall health condition. Their ages varied from 44 to 81 years, body masses from 53.3 to 95.6 kg, heights from 151.5 to 184.0 cm, body-mass indexes (BMI) from 17.5 to 31.5 kg/m^2^, Hoehn & Yahr scale between 1 and 4, and 14 with freezing of gait (FoG). Inclusion criteria were the absence of neurological or physical dysfunctions other than those associated with PD and no diagnosed vestibular, visual, or somatosensory dysfunctions as self-declared.

### Stabilography

The stabilography evaluation was based on the most common practices used in research laboratories (for a review, see Duarte and Freitas, 2010; Scoppa et al., 2013; Paillard and Noe, 2015). We evaluated the patients' balance while standing still for 30 s, in each of four different conditions: on a rigid surface with eyes open; on a rigid surface with eyes closed; on an unstable surface with eyes open; on an unstable surface with eyes closed. Each condition was performed three times, and the order of the conditions was randomized among patients. For the rigid surface conditions, the subjects stood on a 40 × 60 cm force platform (OPT400600-1000; AMTI, Watertown, MA, USA) under the foot, and for the unstable surface conditions, the subjects stood on a 6-cm high foam block (Balance Pad; Airex AG, Sins, Switzerland) placed on the force platform. In all conditions, the patients were required to stand barefoot and as still as possible with their arms at their sides and look at a 5 cm round black target placed on the subject's eye height on a wall 3 m ahead. For the trials where the eyes were kept closed, patients were first instructed to look at the target with eyes open, find a stable and comfortable posture, and close their eyes. A few seconds later, the data acquisition started. For all the trials, the patient's feet were placed with an angle of 20 degrees between them, and their heels were kept 10 cm apart by asking the subjects to stand on lines marked on the top of the force platforms and on the foam blocks (for a review see Santos and Duarte, 2016). The trials were conducted in an 11.5 × 9.3 m room with white walls and adequate illumination. The data acquisition of the GRF was performed at a sampling frequency of 100 Hz with the Cortex software version 7.0 (Motion Analysis, Santa Rosa, CA, USA).

### Protocol

We followed the following procedure for the stabilography (based on Santos and Duarte, 2016):

1. The force platform was zeroed according to the equipment's manuals.
2. The researcher explained to each patient the process of data collection. The patient was informed that he or she would be monitored during the data collection and that there should not be any verbal communication during the trials but that he or she could interrupt the data collection if desired and that assistance would be given if necessary.
3. After these explanations, the patient signed the informed consent form.
4. The researcher interviewed the subject to collect information about his or her clinical data, medication, and diagnosis of the disease.
5. At the beginning of each session, two experienced researchers in movement disorders applied the following scales: UPDRS-II and UPDRS-III (Fahn and Elton, 1987), H&Y (Hoehn and Yahr, 2001), NFOG-Q (Nieuwboer et al., 2009), MoCA (Nasreddine et al., 2005), Mini-BESTest (Horak et al., 2009), and FES-I (Yardley et al., 2005). The assessments of each item on the scales were given by consensus among researchers.
6. Participants rested for 10 min.
7. The researcher instructed the patient how to stand on the force platform according to the task (with open or closed eyes and standing on a rigid or unstable surface). The patient's feet were positioned on the marks at the force platform or on the foam blocks. The researcher instructed the patient to maintain the position of his or her arms along his or her body and to stand as still as possible. During the trials, with the patient's eyes open, the patients were told to fix their gaze ahead on the round black target placed on the wall at the patient's eye level. During the trials with eyes closed, the patients were told to fix their gaze ahead at the same target, close their eyes when they felt ready, and only open them when the researcher indicated that the trial was over.
8. The researcher started the data collection around 5 s later when the patient said he or she was ready.
9. At the end of the trial, the subject was assisted to step from the force platform, and he or she could rest (and sit if desired) for about one minute before the subsequent trial.
10. If the subject was unable to complete a 30 s trial, the test was stopped.

### Data Acquisition and Processing

The force platform data were smoothed with a 10 Hz fourth-order zero-lag low-pass Butterworth filter, and the center of pressure (CoP) was calculated according to the standard formula (Santos and Duarte, 2016). The GRF data (including CoP) was transformed from the coordinate systems of the force platform into the laboratory coordinate system (the coordinate system created by the motion capture system) via transformation matrices using the Cortex software. As a result, the data is presented as the mediolateral, anteroposterior, and vertical components of CoP, force, the moment of force, and the free moment of force. The free moment of force is the moment around the normal to the force plate on the subject's foot.

## Results

All the data is available at Figshare (doi: https://doi.org/10.6084/m9.figshare.13530587); the data is stored in ASCII (text) format with tab-separated columns that can be downloaded as a single compressed file that is made available under the CC0 1.0 license (https://creativecommons.org/publicdomain/zero/1.0/). The data set comprises a file with metadata plus 767 files with the GRF for the 32 patients in each medication condition.

The clinical characteristics of the patients are presented in Table 1.

**Table 1:** Mean (standard deviation) of the characteristics of the participants separately by medication condition.

	**ON**	**OFF**
Disease duration (years)	7.23 (4.49)	
L-Dopa equivalent units (mg·day^−1^)	787.38 (675.07)	
NFoG-Q (score)	6.34 (8.76)	
MoCA (score)	23.47 (3.87)	
H&Y stage (score)	2.31 (0.64)	2.38 (0.61)
UPDRS-II (score)	3.91 (3.06)	5.44 (3.21)
UPDRS-III (score)	18.19 (5.81)	26.41 (11.06)
UPDRS-III rigidity item (score)	4.72 (4.10)	5.69 (3.65)
UPDRS-III gait item (score)	1.09 (0.89)	1.28 (0.77)
Mini-BESTest (score)	26.50 (4.66)	25.06 (5.16)
FES-I (score)	25.72 (8.32)	33.31 (10.01)

*MoCA, Montreal Cognitive Assessment; H&Y, Hoehn & Yahr; NFoG-Q, New Freezing of Gait Questionnaire; UPDRS-II, motor aspects of experiences of daily living Unified Parkinson's disease rating scale; UPDRS-III, motor score Unified Parkinson's disease rating scale (total score and a separate score for items 5—rigidity—and 12—gait); Mini-BESTest, Mini-Test of Balance Assessment System scale; FES-I, Falls Efficacy Scale International*.

### Metadata

The metadata file named PDDSinfo.txt contains 39 information from the anamnesis and clinical scales of each patient. Here is the coding for the metadata:

1. ID: the file name of the stabilography trial (PDPDSXX, in which PDPDS stands for the project's name (Parkinson's Disease Posture Data Set); XX identifies the patient and varies from 01 to 32.
2. Gender: gender (F or M).
3. Age: patient's age in years.
4. Height (cm): height in meters (measured with a calibrated stadiometer).
5. Weight (kg): weight in kilograms (measured with a calibrated scale).
6. BMI (kg/m^2^): body mass index in kg/m^2^.
7. Ortho-Prosthesis: name of the orthosis or prosthesis the subject wears (“No” if the patient did not wear any orthosis or prosthesis).
8. Years of formal study: years of formal education.
9. Disease duration (years): year from diagnosis.
10. L-Dopa equivalent units (mg•day^−1^): total daily levodopa equivalent dose in mg•day^−1^ according to Tomlinson et al. (2010).
11. FoG group: presence (freezers) or not (non-freezers) of freezing of gait.
12. NFoG-Q (score): score of New Freezing of Gait Questionnaire.
13. PIGD or TD: Postural Instability/Gait Difficulty (PIGD) or Tremor Dominant (TD) phenotypes of Parkinson's disease according to Stebbins et al. (2013).
14. Initial symptoms: self-reported initial symptoms.
15. Is there a family history of PD? Who?
16. Do you feel improvement after using the antiparkinsonian medicine?: Yes or No.
17. Have you ever had any surgery? Which?
18. Any rehabilitation or physical activity?: name of the rehabilitation or physical activity performed by the patient (“No” if the patient did not perform any rehabilitation or physical activity).
19. Other diseases (cardiovascular, bone, etc.): name of the disability of the patient (“No” if the patient did not present any disability).
20. ON—Hoehn & Yahr: Hoehn & Yahr score in the ON medication.
21. ON—MoCA: MoCA score in the ON medication.
22. ON—UPDRS-II: total score of the UPDRS-II in the ON medication.
23. ON—UPDRS-III: total score of the UPDRS-III in the ON medication.
24. ON—UPDRS-III—Rigidity: score of item 5—rigidity of UPDRS-III in the ON medication.
25. ON—UPDRS-III—Gait: score of item 12—rigidity of UPDRS-III in the ON medication.
26. ON—UPDRS-III—Bradykinesia: score of item 14—bradykinesia of UPDRS-III in the ON medication.
27. ON—UPDRS-III—Dyskinesia: score of item 15—dyskinesia of UPDRS-III in the ON medication.
28. ON—miniBESTest: miniBESTest score in the ON medication.
29. ON—FES-I: FES-I score in the ON medication.
30. OFF—Hoehn & Yahr: Hoehn & Yahr score in the OFF medication.
31. OFF—MoCA: MoCA score in the OFF medication.
32. OFF—UPDRS-II: total score of the UPDRS-II in the OFF medication.
33. OFF—UPDRS-III: total score of the UPDRS-III in the OFF medication.
34. OFF—UPDRS-III—Rigidity: score of item 5—rigidity of UPDRS-III in the OFF medication.
35. OFF—UPDRS-III—Gait: score of item 12—rigidity of UPDRS-III in the OFF medication.
36. OFF—UPDRS-III—Bradykinesia: score of item 14—bradykinesia of UPDRS-III in the OFF medication.
37. OFF—UPDRS-III—Dyskinesia: score of item 15—dyskinesia of UPDRS-III in the OFF medication.
38. OFF—miniBESTest: miniBESTest score in the OFF medication.
39. OFF—FES-I: FES-I score in the O OFF medication.

### Ground Reaction Force Data

Each text file with the GRF data is named by the corresponding condition and trial (PDPDSXX_MED_COND_TRIAL.txt, XX identifies the patient and varies from 01 to 32; MED identifies the medication condition and can be ON or OFF; COND identifies the stabilography condition and is either RS_EO, RS_EC, US_EO or US_EC, with RS rigid surface, US unstable surface, EO eyes open and EC eyes closed; and Z identifies the number of repetitions and varies from 1 to 3). The header refers to the signal at each column and are:

1. Time: Time in s.
2. GRFml [N]: mediolateral Ground Reaction Force (GRF) in N.
3. GRFap [N]: anteroposterior GRF in N.
4. GRFv [N]: vertical GRF in N.
5. Mml [N.m]: mediolateral moment of force in N.m.
6. Map [N.m]: anteroposterior moment of force in N.m.
7. Mml [N.m]: vertical moment of force in N.m.
8. Mfree [N.m]: Free Moment at the direction Y in Nm.
9. CoPml [m]: mediolateral center of pressure in m.
10. CoPap [m]: anteroposterior center of pressure in m.

## Discussion

This study provides a publicly available data set with qualitative and quantitative evaluations related to the balance of PD patients. All the data is available at Figshare (doi: https://doi.org/10.6084/m9.figshare.13530587).

These measures are relevant for the scientific community, considering the current research on balance in people with Parkinson's disease found in the literature, especially about the involvement of dopaminergic medication on the motor and cognitive aspects of these patients, and muscle weakness especially in the subtype characterized by PIGD.

We hope that the availability of this set of public data will contribute to research on postural control in PD patients since this topic is highly relevant, both for the pathophysiological and biomechanical understanding of the imbalances that affect this population, as well as for the evolution of therapeutic strategies, which still lack effective responses.

## Data Availability Statement

The datasets presented in this study can be found in online repositories. The names of the repository/repositories and accession number(s) can be found in the article/supplementary material.

## Ethics Statement

The studies involving human participants were reviewed and approved by Federal University of ABC. The patients/participants provided their written informed consent to participate in this study.

## Author Contributions

CO: conceptualization, methodology, data collect, writing—original draft, and writing—review and editing. CR: conceptualization, methodology, data collect, and writing—original draft. RT, SH, EL, CB, TS, LS, TN, DC, and EG: methodology and data collect. MC: methodology, data collect, and writing—review and editing. DBC: conceptualization, methodology, data collect, writing—original draft, writing—review and editing, and supervision. All authors contributed to the article and approved the submitted version.

## Funding

This study was supported by Fundação de Amparo à Pesquisa do Estado de São Paulo (FAPESP/Brazil)—grant #2019/06604-1, Universidade Federal do ABC (UFABC/Brazil), and by Coordenação de Aperfeiçoamento de Pessoal de Nível Superior (CAPES/Brazil).

## Conflict of Interest

The authors declare that the research was conducted in the absence of any commercial or financial relationships that could be construed as a potential conflict of interest.

## Publisher's Note

All claims expressed in this article are solely those of the authors and do not necessarily represent those of their affiliated organizations, or those of the publisher, the editors and the reviewers. Any product that may be evaluated in this article, or claim that may be made by its manufacturer, is not guaranteed or endorsed by the publisher.

## References

[B1] Baston C. Mancini M. Rocchi L. Horak F. (2016). Effects of levodopa on postural strategies in Parkinson's disease. Gait Posture 46, 26–29. 10.1016/j.gaitpost.2016.02.009 27131172 PMC5321663

[B2] Beuter A. Hernandez R. Rigal R. Modolo J. Blanchet P. J. (2008). Postural sway and effect of levodopa in early Parkinson's disease. Can. J. Neurol. Sci. 35, 65–68. 10.1017/S0317167100007575 18380279

[B3] Chastan N. Debono B. Maltete D. Weber J. (2008). Discordance between measured postural instability and absence of clinical symptoms in Parkinson's disease patients in the early stages of the disease. Mov. Disord. 23, 366–372. 10.1002/mds.21840 18044726

[B4] Chung K. A. Lobb B. M. Nutt J. G. Mcnames J. Horak F. (2010). Objective measurement of dyskinesia in Parkinson's disease using a force plate. Mov Disord. 25, 602–608. 10.1002/mds.22856 20213818 PMC3148105

[B5] Curtze C. Nutt J. G. Carlson-Kuhta P. Mancini M. Horak F. B. (2015). Levodopa is a double-edged sword for balance and gait in people with Parkinson's disease. Mov. Disord. 30, 1361–1370. 10.1002/mds.26269 26095928 PMC4755510

[B6] Dos Santos D. A. Fukuchi C. A. Fukuchi R. K. Duarte M. (2017). A data set with kinematic and ground reaction forces of human balance. PeerJ 5, e3626. 10.7717/peerj.3626 28761798 PMC5534162

[B7] Duarte M. Freitas S. M. (2010). Revision of posturography based on force plate for balance evaluation. Braz. J. Phys. Ther. 14, 183–192. 10.1590/S1413-35552010000300003 20730361

[B8] Fahn S. Elton R. L. (1987). “Unified Parkinson's disease rating scale,” in Recent Developments in Parkinson's Disease, eds S. Fahn, C. D. Marsden, M. Goldstein, and D. B. Calne (Florham Park, NJ: Macmillan Health Care Information), 232.

[B9] Feller K. J. Peterka R. J. Horak F. B. (2019). Sensory re-weighting for postural control in Parkinson's disease. Front. Hum. Neurosci. 13, 126. 10.3389/fnhum.2019.00126 31057379 PMC6478764

[B10] Hoehn M. M. Yahr M. D. (2001). Parkinsonism: onset, progression, and mortality. Neurology 57, S11–S26. 10.1212/wnl.17.5.427 11775596

[B11] Horak F. B. Wrisley D. M. Frank J. (2009). The Balance Evaluation Systems Test (BESTest) to differentiate balance deficits. Phys Ther 89, 484–498. 10.2522/ptj.20080071 19329772 PMC2676433

[B12] Mancini M. Carlson-Kuhta P. Zampieri C. Nutt J. G. Chiari L. Horak F. B. (2012). Postural sway as a marker of progression in Parkinson's disease: a pilot longitudinal study. Gait Posture 36, 471–476. 10.1016/j.gaitpost.2012.04.010 22750016 PMC3894847

[B13] Mancini M. Horak F. B. Zampieri C. Carlson-Kuhta P. Nutt J. G. Chiari L. (2011). Trunk accelerometry reveals postural instability in untreated Parkinson's disease. Parkinsonism Relat. Disord. 17, 557–562. 10.1016/j.parkreldis.2011.05.010 21641263 PMC5327861

[B14] Nantel J. Mcdonald J. C. Bronte-Stewart H. (2012). Effect of medication and STN-DBS on postural control in subjects with Parkinson's disease. Parkinsonism Relat. Disord. 18, 285–289. 10.1016/j.parkreldis.2011.11.005 22130147

[B15] Nasreddine Z. S. Phillips N. A. Bedirian V. Charbonneau S. Whitehead V. Collin I. . (2005). The montreal cognitive assessment, MoCA: a brief screening tool for mild cognitive impairment. J. Am. Geriatr. Soc. 53, 695–699. 10.1111/j.1532-5415.2005.53221.x 15817019

[B16] Nieuwboer A. Rochester L. Muncks L. Swinnen S. P. (2009). Motor learning in Parkinson's disease: limitations and potential for rehabilitation. Parkinsonism Relat. Disord. 15(Suppl. 3), S53–S58. 10.1016/S1353-8020(09)70781-3 20083008

[B17] Paillard T. Noe F. (2015). Techniques and methods for testing the postural function in healthy and pathological subjects. Biomed. Res. Int. 2015, 891390. 10.1155/2015/891390 26640800 PMC4659957

[B18] Peterson D. S. Horak F. B. (2016). The effect of levodopa on improvements in protective stepping in people with Parkinson's disease. Neurorehabil. Neural Repair. 30, 931–940. 10.1177/1545968316648669 27162165 PMC5048496

[B19] Rocchi L. Chiari L. Horak F. B. (2002). Effects of deep brain stimulation and levodopa on postural sway in Parkinson's disease. J. Neurol. Neurosurg. Psychiatry 73, 267–274. 10.1136/jnnp.73.3.267 12185157 PMC1738049

[B20] Santos D. A. Duarte M. (2016). A public data set of human balance evaluations. PeerJ 4, e2648. 10.7717/peerj.2648 27833813 PMC5101613

[B21] Scoppa F. Capra R. Gallamini M. Shiffer R. (2013). Clinical stabilometry standardization: basic definitions–acquisition interval–sampling frequency. Gait Posture 37, 290–292. 10.1016/j.gaitpost.2012.07.009 22889928

[B22] Stebbins G. T. Goetz C. G. Burn D. J. Jankovic J. Khoo T. K. Tilley B. C. (2013). How to identify tremor dominant and postural instability/gait difficulty groups with the movement disorder society unified Parkinson's disease rating scale: comparison with the unified Parkinson's disease rating scale. Mov. Disord. 28, 668–670. 10.1002/mds.25383 23408503

[B23] Stylianou A. P. Mcvey M. A. Lyons K. E. Pahwa R. Luchies C. W. (2011). Postural sway in patients with mild to moderate Parkinson's disease. Int. J. Neurosci. 121, 614–621. 10.3109/00207454.2011.602807 21740307

[B24] Tomlinson C. L. Stowe R. Patel S. Rick C. Gray R. Clarke C. E. (2010). Systematic review of levodopa dose equivalency reporting in Parkinson's disease. Mov. Disord. 25, 2649–2653. 10.1002/mds.23429 21069833

[B25] Yardley L. Beyer N. Hauer K. Kempen G. Piot-Ziegler C. Todd C. (2005). Development and initial validation of the Falls Efficacy Scale-International (FES-I). Age Ageing 34, 614–619. 10.1093/ageing/afi196 16267188

